# *N*′-Hy­droxy­pyridine-2-carboximidamide–succinic acid (2/1)

**DOI:** 10.1107/S1600536813014359

**Published:** 2013-05-31

**Authors:** Jiyong Liu

**Affiliations:** a Department of Chemistry, Zhejiang University, Hangzhou, Zhejiang 310027, People’s Republic of China

## Abstract

The asymmetric unit of the title co-crystal, C_6_H_7_N_3_O·0.5C_4_H_6_O_4_, comprises one *N*′-hy­droxy­pyridine-2-carboximidamide mol­ecule and half a succinic acid mol­ecule (the whole molecule is generated by inversion symmetry). In the crystal, mol­ecules are assembled into columns along [110], *via* strong N—H⋯O, O—H⋯O and O—H⋯N hydrogen bonds.

## Related literature

For background to cocrystals and their applications, see: Biradha *et al.* (2009 ▶); Desiraju (1995 ▶, 2003 ▶).
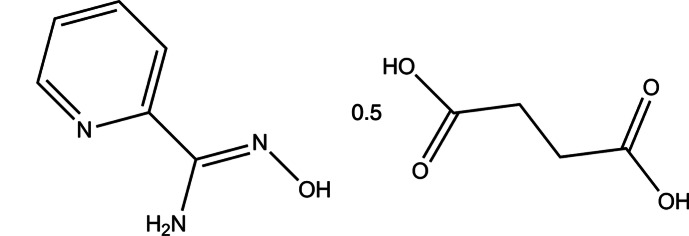


## Experimental

### 

#### Crystal data

C_6_H_7_N_3_O·0.5C_4_H_6_O_4_
*M* *_r_* = 196.19
Monoclinic, 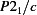

*a* = 8.6707 (8) Å
*b* = 5.2628 (4) Å
*c* = 20.6693 (15) Å
β = 93.014 (7)°
*V* = 941.87 (13) Å^3^
*Z* = 4
Mo *K*α radiation
μ = 0.11 mm^−1^
*T* = 293 K
0.32 × 0.28 × 0.15 mm

#### Data collection

Oxford Diffraction Xcalibur (Atlas, Gemini ultra) diffractometer
Absorption correction: multi-scan (*CrysAlis PRO*; Oxford Diffraction, 2009 ▶) *T*_min_ = 0.966, *T*_max_ = 0.984
4220 measured reflections
1733 independent reflections
1255 reflections with *I* > 2σ(*I*)
*R*_int_ = 0.029

#### Refinement

*R*[*F* ^2^ > 2σ(*F* ^2^)] = 0.039
*wR*(*F* ^2^) = 0.106
*S* = 1.05
1733 reflections
140 parameters
4 restraints
H atoms treated by a mixture of independent and constrained refinement
Δρ_max_ = 0.16 e Å^−3^
Δρ_min_ = −0.15 e Å^−3^

### 

Data collection: *CrysAlis PRO* (Oxford Diffraction, 2009 ▶); cell refinement: *CrysAlis PRO*; data reduction: *CrysAlis PRO*; program(s) used to solve structure: *SHELXS97* (Sheldrick, 2008 ▶); program(s) used to refine structure: *SHELXL97* (Sheldrick, 2008 ▶); molecular graphics: *OLEX2* (Dolomanov *et al.*, 2009 ▶); software used to prepare material for publication: *OLEX2*.

## Supplementary Material

Crystal structure: contains datablock(s) I, global. DOI: 10.1107/S1600536813014359/bg2507sup1.cif

Structure factors: contains datablock(s) I. DOI: 10.1107/S1600536813014359/bg2507Isup2.hkl

Supplementary material file. DOI: 10.1107/S1600536813014359/bg2507Isup3.cml

Additional supplementary materials:  crystallographic information; 3D view; checkCIF report

## Figures and Tables

**Table 1:** Hydrogen-bond geometry (Å, °)

*D*—H⋯*A*	*D*—H	H⋯*A*	*D*⋯*A*	*D*—H⋯*A*
O3—H3*A*⋯N3	0.84 (1)	1.80 (1)	2.6362 (18)	175 (2)
O1—H1⋯O2	0.83 (1)	1.96 (1)	2.7608 (18)	164 (2)
N2—H2*B*⋯O1^i^	0.86 (1)	2.26 (1)	3.025 (2)	149 (2)

Symmetry code: (i) 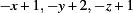.

**Fig. 1.**
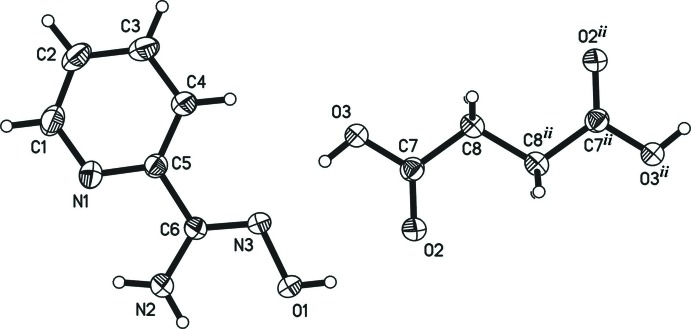
ORTEP view of the title compound. The displacement ellipsoids are drawn at 30% probability level. Symmetry code: (ii) -x, -y, -z+1.

**Fig. 2.**
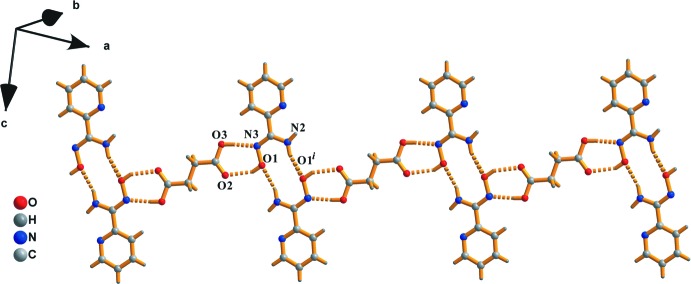
The one-dimensional chain of the compound along [110] direction. Symmetry code: (i) -x+1, -y+2, -z+1

## supplementary crystallographic information

### Comment

There has been an instense interest in the preparation of cocrystals which is evident from the increasing number of research publications on this topic in recent years. With reliable strategies, cocrystals could offer a modular approach to delvelping materials with desirable properties.(Desiraju, 1995, 2003; Biradha *et al.*, 2009) Cocrystals are created by utilizing weak noncovalent interactions such as hydrogen bonds. Herein we report the structure of the first cocrystal of the pyC(NH_2_)NOH molecule.

The asymmetric unit of the title compound (Fig.1) contains one pyC(NH_2_)NOH molecule and one half succinic acid molecule (the entire molecule is completed by the application of a centre of inversion). The pyridine rings and the N2—C6—N3—O1 rings are nearly coplanar, and the C7—C8—C8^ii^—C7^ii^ torsion angle [Symmetry codes: (ii)-*x*, -*y*, -*z* + 1] of succinic acid is 180° restricted by crystallographic centrosymmetry. The proton of the carboxylate O atom (O3) of the succinic acid molecule forms a strong hydrogen bond with atom N3 of the pyC(NH_2_)NOH molecule, at the same time, hydrogen bonding exist between hydroxyl O1 and carboxylater O2 atoms.(see Table 1 for hydrogen bond geometry). In addition, strong intermolecular N2—H2B···O1^i^ [Symmetry codes: (i)-*x* + 1, -*y* + 2, -*z* + 1] hydrogen bonding supplement intermolecular O—H···O and O—H···N hydrogen bonding to form columns running parallel to the [110] direction. (Fig 2)

### Experimental

A stoichiometric amount in the ratio of 2:1 of pyC(CH_2_)NOH and succinic acid were dissolved in 20 ml e thanol, and the solution slowly left to evaporate to afford colourless block-like crystals after one week.

### Refinement

H atoms bonded to C atoms were placed in geometrically calculated positions and were refined using a riding model, with *U*_iso_(H) = 1.2 *U*_eq_(C). The N-bound and O-bound H atoms were located in the difference map and coordinates refined freely together with their isotropic displacement parameters.

### Crystal data

**Table:**

C_6_H_7_N_3_O·0.5C_4_H_6_O_4_	*F*(000) = 412
*M**_r_* = 196.19	*D*_x_ = 1.384 Mg m^−^^3^
Monoclinic, *P*2_1_/*c*	Mo *K*α radiation, λ = 0.71073 Å
Hall symbol: -P 2ybc	Cell parameters from 1508 reflections
*a* = 8.6707 (8) Å	θ = 3.0–29.6°
*b* = 5.2628 (4) Å	µ = 0.11 mm^−^^1^
*c* = 20.6693 (15) Å	*T* = 293 K
β = 93.014 (7)°	Block, colourless
*V* = 941.87 (13) Å^3^	0.32 × 0.28 × 0.15 mm
*Z* = 4	

### Data collection

**Table:**

Oxford Diffraction Xcalibur (Atlas, Gemini ultra) diffractometer	1733 independent reflections
Radiation source: fine-focus sealed tube	1255 reflections with *I* > 2σ(*I*)
Graphite monochromator	*R*_int_ = 0.029
Detector resolution: 10.3592 pixels mm^-1^	θ_max_ = 25.4°, θ_min_ = 3.0°
ω scans	*h* = −10→10
Absorption correction: multi-scan (*CrysAlis PRO*; Oxford Diffraction, 2009)	*k* = −5→6
*T*_min_ = 0.966, *T*_max_ = 0.984	*l* = −24→20
4220 measured reflections	

### Refinement

**Table:**

Refinement on *F*^2^	Secondary atom site location: difference Fourier map
Least-squares matrix: full	Hydrogen site location: inferred from neighbouring sites
*R*[*F*^2^ > 2σ(*F*^2^)] = 0.039	H atoms treated by a mixture of independent and constrained refinement
*wR*(*F*^2^) = 0.106	*w* = 1/[σ^2^(*F*_o_^2^) + (0.0475*P*)^2^ + 0.0833*P*] where *P* = (*F*_o_^2^ + 2*F*_c_^2^)/3
*S* = 1.05	(Δ/σ)_max_ < 0.001
1733 reflections	Δρ_max_ = 0.16 e Å^−^^3^
140 parameters	Δρ_min_ = −0.15 e Å^−^^3^
4 restraints	Extinction correction: *SHELXL97* (Sheldrick, 2008), Fc^*^=kFc[1+0.001xFc^2^λ^3^/sin(2θ)]^-1/4^
Primary atom site location: structure-invariant direct methods	Extinction coefficient: 0.106 (6)

### Special details

**Table:**

Geometry. All e.s.d.'s (except the e.s.d. in the dihedral angle between two l.s. planes) are estimated using the full covariance matrix. The cell e.s.d.'s are taken into account individually in the estimation of e.s.d.'s in distances, angles and torsion angles; correlations between e.s.d.'s in cell parameters are only used when they are defined by crystal symmetry. An approximate (isotropic) treatment of cell e.s.d.'s is used for estimating e.s.d.'s involving l.s. planes.
Refinement. Refinement of *F*^2^ against ALL reflections. The weighted *R*-factor *wR* and goodness of fit *S* are based on *F*^2^, conventional *R*-factors *R* are based on *F*, with *F* set to zero for negative *F*^2^. The threshold expression of *F*^2^ > σ(*F*^2^) is used only for calculating *R*-factors(gt) *etc*. and is not relevant to the choice of reflections for refinement. *R*-factors based on *F*^2^ are statistically about twice as large as those based on *F*, and *R*- factors based on ALL data will be even larger.

### Fractional atomic coordinates and isotropic or equivalent isotropic displacement parameters (Å^2^)

**Table:**

	*x*	*y*	*z*	*U*_iso_*/*U*_eq_	
O1	0.39704 (16)	0.7215 (3)	0.47132 (6)	0.0554 (4)	
H1	0.352 (3)	0.597 (3)	0.4861 (11)	0.083*	
O2	0.19671 (16)	0.3449 (3)	0.50695 (6)	0.0595 (4)	
O3	0.11775 (15)	0.3628 (3)	0.40307 (6)	0.0527 (4)	
H3A	0.182 (2)	0.482 (3)	0.4024 (11)	0.079*	
N1	0.36547 (19)	1.1544 (3)	0.27768 (7)	0.0536 (5)	
N2	0.4806 (2)	1.0874 (3)	0.39785 (8)	0.0546 (5)	
H2A	0.497 (2)	1.209 (3)	0.3713 (8)	0.066*	
H2B	0.517 (2)	1.079 (4)	0.4370 (6)	0.066*	
N3	0.32452 (17)	0.7305 (3)	0.40829 (6)	0.0429 (4)	
C1	0.3146 (3)	1.1925 (4)	0.21617 (10)	0.0630 (6)	
H1A	0.3535	1.3306	0.1942	0.076*	
C2	0.2086 (3)	1.0397 (4)	0.18374 (10)	0.0623 (6)	
H2	0.1772	1.0720	0.1408	0.075*	
C3	0.1503 (3)	0.8383 (4)	0.21632 (10)	0.0691 (7)	
H3	0.0768	0.7322	0.1961	0.083*	
C4	0.2017 (3)	0.7947 (4)	0.27915 (10)	0.0589 (6)	
H4	0.1639	0.6578	0.3020	0.071*	
C5	0.30971 (19)	0.9554 (3)	0.30816 (8)	0.0394 (4)	
C6	0.37353 (19)	0.9207 (3)	0.37593 (8)	0.0375 (4)	
C7	0.1144 (2)	0.2691 (3)	0.46188 (8)	0.0404 (4)	
C8	−0.0020 (2)	0.0614 (3)	0.46712 (8)	0.0450 (5)	
H8A	0.0166	−0.0675	0.4349	0.054*	
H8B	−0.1043	0.1305	0.4575	0.054*	

### Atomic displacement parameters (Å^2^)

**Table:**

	*U*^11^	*U*^22^	*U*^33^	*U*^12^	*U*^13^	*U*^23^
O1	0.0678 (9)	0.0588 (9)	0.0376 (7)	−0.0209 (7)	−0.0147 (6)	0.0097 (6)
O2	0.0687 (9)	0.0703 (9)	0.0387 (7)	−0.0310 (8)	−0.0055 (7)	0.0058 (7)
O3	0.0573 (9)	0.0596 (9)	0.0402 (7)	−0.0211 (7)	−0.0069 (6)	0.0095 (7)
N1	0.0545 (10)	0.0596 (10)	0.0466 (10)	−0.0103 (8)	0.0023 (8)	0.0131 (8)
N2	0.0664 (11)	0.0567 (11)	0.0400 (9)	−0.0249 (9)	−0.0048 (8)	0.0050 (8)
N3	0.0482 (9)	0.0450 (9)	0.0347 (8)	−0.0081 (7)	−0.0070 (6)	0.0036 (7)
C1	0.0607 (13)	0.0774 (15)	0.0508 (13)	−0.0053 (12)	0.0014 (10)	0.0252 (11)
C2	0.0659 (14)	0.0788 (15)	0.0410 (11)	0.0078 (12)	−0.0075 (10)	0.0142 (11)
C3	0.0829 (16)	0.0678 (14)	0.0534 (13)	−0.0107 (12)	−0.0279 (12)	0.0085 (11)
C4	0.0737 (14)	0.0512 (12)	0.0496 (12)	−0.0162 (10)	−0.0176 (10)	0.0112 (10)
C5	0.0412 (10)	0.0391 (10)	0.0378 (10)	0.0007 (8)	0.0011 (8)	0.0016 (8)
C6	0.0390 (10)	0.0371 (9)	0.0365 (9)	−0.0042 (8)	0.0016 (7)	−0.0013 (8)
C7	0.0411 (10)	0.0433 (10)	0.0370 (10)	0.0000 (8)	0.0024 (8)	0.0003 (8)
C8	0.0432 (10)	0.0467 (11)	0.0448 (10)	−0.0094 (8)	0.0002 (8)	0.0020 (8)

### Geometric parameters (Å, º)

**Table:**

O1—N3	1.4173 (17)	C1—H1A	0.9300
O1—H1	0.828 (10)	C2—C3	1.367 (3)
O2—C7	1.211 (2)	C2—H2	0.9300
O3—C7	1.314 (2)	C3—C4	1.370 (3)
O3—H3A	0.840 (10)	C3—H3	0.9300
N1—C5	1.327 (2)	C4—C5	1.376 (3)
N1—C1	1.339 (2)	C4—H4	0.9300
N2—C6	1.339 (2)	C5—C6	1.490 (2)
N2—H2A	0.859 (9)	C7—C8	1.495 (2)
N2—H2B	0.856 (9)	C8—C8^i^	1.503 (3)
N3—C6	1.288 (2)	C8—H8A	0.9700
C1—C2	1.370 (3)	C8—H8B	0.9700
			
N3—O1—H1	99.8 (16)	C3—C4—H4	120.3
C7—O3—H3A	110.1 (16)	C5—C4—H4	120.3
C5—N1—C1	117.31 (17)	N1—C5—C4	122.36 (16)
C6—N2—H2A	114.0 (14)	N1—C5—C6	114.61 (15)
C6—N2—H2B	120.2 (14)	C4—C5—C6	123.04 (16)
H2A—N2—H2B	125 (2)	N3—C6—N2	125.12 (16)
C6—N3—O1	111.14 (13)	N3—C6—C5	117.83 (14)
N1—C1—C2	123.79 (19)	N2—C6—C5	117.03 (15)
N1—C1—H1A	118.1	O2—C7—O3	123.17 (16)
C2—C1—H1A	118.1	O2—C7—C8	123.90 (16)
C3—C2—C1	118.03 (18)	O3—C7—C8	112.93 (15)
C3—C2—H2	121.0	C7—C8—C8^i^	113.34 (18)
C1—C2—H2	121.0	C7—C8—H8A	108.9
C2—C3—C4	119.1 (2)	C8^i^—C8—H8A	108.9
C2—C3—H3	120.4	C7—C8—H8B	108.9
C4—C3—H3	120.4	C8^i^—C8—H8B	108.9
C3—C4—C5	119.35 (19)	H8A—C8—H8B	107.7

Symmetry code: (i) −*x*, −*y*, −*z*+1.

### Hydrogen-bond geometry (Å, º)

**Table:**

*D*—H···*A*	*D*—H	H···*A*	*D*···*A*	*D*—H···*A*
O3—H3*A*···N3	0.84 (1)	1.80 (1)	2.6362 (18)	175 (2)
O1—H1···O2	0.83 (1)	1.96 (1)	2.7608 (18)	164 (2)
N2—H2*B*···O1^ii^	0.86 (1)	2.26 (1)	3.025 (2)	149 (2)

Symmetry code: (ii) −*x*+1, −*y*+2, −*z*+1.
